# BAFF system expression in double negative 2, activated naïve and activated memory B cells in systemic lupus erythematosus

**DOI:** 10.3389/fimmu.2023.1235937

**Published:** 2023-08-22

**Authors:** Jhonatan Antonio Álvarez Gómez, Diana Celeste Salazar-Camarena, Ilce Valeria Román-Fernández, Pablo César Ortiz-Lazareno, Alvaro Cruz, José Francisco Muñoz-Valle, Miguel Marín-Rosales, Noemí Espinoza-García, Nefertari Sagrero-Fabela, Claudia Azucena Palafox-Sánchez

**Affiliations:** ^1^ Doctorado en Ciencias en Biología Molecular en Medicina (DCBMM), Centro Universitario de Ciencias de la Salud, Universidad de Guadalajara, Guadalajara, Jalisco, Mexico; ^2^ Grupo de Inmunología Molecular, Centro Universitario de Ciencias de la Salud, Universidad de Guadalajara, Guadalajara, Jalisco, Mexico; ^3^ Instituto de Investigación en Ciencias Biomédicas (IICB), Centro Universitario de Ciencias de la Salud, Universidad de Guadalajara, Guadalajara, Jalisco, Mexico; ^4^ División de Inmunología, Centro de Investigación Biomédica de Occidente (CIBO), Instituto Mexicano del Seguro Social (IMSS), Guadalajara, Jalisco, Mexico; ^5^ Hospital General de Occidente, Secretaría de Salud Jalisco, Guadalajara, Jalisco, Mexico; ^6^ Doctorado en Ciencias Biomédicas (DCB), Centro Universitario de Ciencias de la Salud, Universidad de Guadalajara, Guadalajara, Jalisco, Mexico

**Keywords:** DN2, aNAV, memory B cells, BAFF system expression, atypical B cells, SLE

## Abstract

**Introduction:**

B cell activating factor (BAFF) has an important role in normal B cell development. The aberrant expression of BAFF is related with the autoimmune diseases development like Systemic Lupus Erythematosus (SLE) for promoting self-reactive B cells survival. BAFF functions are exerted through its receptors BAFF-R (BR3), transmembrane activator calcium modulator and cyclophilin ligand interactor (TACI) and B cell maturation antigen (BCMA) that are reported to have differential expression on B cells in SLE. Recently, atypical B cells that express CD11c have been associated with SLE because they are prone to develop into antibody-secreting cells, however the relationship with BAFF remains unclear. This study aims to analyze the BAFF system expression on CXCR5^-^ CD11c^+^ atypical B cell subsets double negative 2 (DN2), activated naïve (aNAV), switched memory (SWM) and unswitched memory (USM) B cells.

**Methods:**

Forty-five SLE patients and 15 healthy subjects (HS) were included. Flow cytometry was used to evaluate the expression of the receptors in the B cell subpopulations. Enzyme-linked immunosorbent assay (ELISA) was performed to quantify the soluble levels of BAFF (sBAFF) and IL-21.

**Results:**

We found increased frequency of CXCR5^-^ CD11c^+^ atypical B cell subpopulations DN2, aNAV, SWM and USM B cells in SLE patients compared to HS. SLE patients had increased expression of membrane BAFF (mBAFF) and BCMA receptor in classic B cell subsets (DN, NAV, SWM and USM). Also, the CXCR5^+^ CD11c^-^ DN1, resting naïve (rNAV), SWM and USM B cell subsets showed higher mBAFF expression in SLE. CXCR5^-^ CD11c^+^ atypical B cell subpopulations DN2, SWM and USM B cells showed strong correlations with the expression of BAFF receptors. The atypical B cells DN2 in SLE showed significant decreased expression of TACI, which correlated with higher IL-21 levels. Also, lower expression of TACI in atypical B cell DN2 was associated with high disease activity.

**Discussion:**

These results suggest a participation of the BAFF system in CXCR5^-^ CD11c^+^ atypical B cell subsets in SLE patients. Decreased TACI expression on atypical B cells DN2 correlated with high disease activity in SLE patients supporting the immunoregulatory role of TACI in autoimmunity.

## Introduction

Systemic lupus erythematosus (SLE) is an autoimmune disease characterized by hyperactive B cells and autoantibody production (1). BAFF (B cell-activating factor) is a type II membrane-bound protein that has a relevant role in SLE pathogenesis promoting self-reactive B cells survival. BAFF can be cleaved and released as a soluble BAFF homotrimer by furin proteases. High levels of soluble BAFF (sBAFF) have been found in SLE patients, correlated with disease activity and anti-dsDNA antibodies (2–4). BAFF effects are performed through its receptors: BR3 (BAFF-R, BAFF receptor), TACI (transmembrane activator calcium modulator and cyclophilin ligand interactor) and BCMA (B cell maturation antigen). BAFF induces B cell survival, T-independent class switch and plasma cells (PC) maintenance; it also can act as a negative regulator of B cell numbers (5). The expression of BAFF receptors on B cells in SLE patients have been evaluated previously showing a decreased expression of BR3 and BCMA associated with higher disease activity (6), highlighting the BAFF receptor’s role in the regulation of B cell homeostasis.

Recently, new B cell subpopulations have been described that are prone to differentiate into PC in an extrafollicular manner and are expanded in SLE (7, 8). These autoimmunity-associated B cells (ABC) could be key in SLE pathogenesis because they are prone to develop into antibody-secreting cells (ASC). Although, T-cell interaction is necessary to initiate this extrafollicular B cell subset, once it is formed, cytokine stimulation is sufficient to drive its development into ASC (8–11). Different expression patterns have been identified in these ABCs like the low levels of CD21 and high levels of CD11c, negative regulators as FcRL5 and transcriptional factors as T-bet, Zeb2 and Blimp-1 (7, 8). Previous studies have classified these ABCs as atypical memory B cells for the presence of somatic hypermutation although they had mixed expression of CD27 (12–14). Other studies have used the CXCR5 and CD11c markers to identify these ABCs as double negative 2 (DN2) B cells (CD27^-^ IgD^-^ CXCR5^-^ CD11c^+^) and activated naïve (aNAV) B cells (CD27^-^ IgD^+^ CXCR5^-^ CD11c^+^). aNAV B cells are precursors of DN2 B cells and differentiate into PC through IL-21, IFN-γ and TLR7 stimulation (7, 15). Although it has been described the important role of BAFF in B cell development, just a few studies have evaluated the presence of the BAFF and their receptors in CXCR5^-^ CD11c^+^ atypical B cell subsets in mice and humans (16, 17). This study aims to analyze the BAFF system expression on CXCR5^-^ CD11c^+^ atypical B cell subpopulations in SLE patients.

## Materials and methods

### SLE patients and healthy subjects

Forty-five SLE patients were included from the Rheumatology department of the Hospital General de Occidente, Guadalajara, Mexico. All patients fulfilled the EULAR/ACR 2019 classification criteria for SLE (18). The Mexican version of the Systemic Lupus Erythematosus Disease Activity Index (MexSLEDAI) score (19) was determined in SLE patients at the time of inclusion. All patients were stratified according to the MexSLEDAI index as follows: 0-2pts Non-Active, 3-5pts Low Disease Activity (LDA) and ≥6pts High Disease Activity (HDA) (20). Also, 15 healthy subjects (HS) with no infections, allergy, or chronic diseases, were included as controls. The ethics in research committee of the hospital approved this study with the registration number CEI-147/21. All the subjects signed an informed consent according to the regulations of the General Health Law on Research for Health in Mexico. The study was conducted according to the ethical principles for medical research involving human subjects established in the Declaration of Helsinki.

### Flow cytometry

Human peripheral blood mononuclear cells (PBMCs) were isolated from fresh peripheral blood with Histopaque-1077 (Merk KGaA; Darmstadt, Germany) by density gradient, and stained using standard flow cytometry methodology with the antibodies described in the **Supplementary Table 1**. Flow cytometric analyses were performed with an Attune Nxt instrument (Thermo Fisher Scientific Inc; Waltham, MA, USA). The compensation was performed with the UltraComp eBeads (CAT: 01-2222-41, Thermo Fisher Scientific Inc; Waltham, MA, USA). Fluorescence minus one (FMO) control was used to adjust the background fluorescence and gates. The histograms of these controls are included in the **Supplementary Figure 1**. The acquisition data was obtained with Attune NxT Software version 3.1.2. The results are reported as percentage (%) of expression and geometric mean fluorescence intensity (MFI). Data were analyzed using FlowJo v.10 (BD, Franklin Lakes, NJ, USA). Instrument quality control was performed once a week during the experiment period using Attune Performance Tracking Beads (CAT: 449754, Thermo Fisher Scientific Inc; Waltham, MA, USA) and settings were maintained for all experiments.

### Cytokine determination

Serum was separated from the total blood of each subject at the time they were involved in the study and were stored at -20°C. Serum cytokine levels of BAFF and IL-21 were quantified in SLE patients and HS by Enzyme-Linked immunosorbent Assay (ELISA) according to the manufacturer’s instructions. Soluble BAFF was measured with Quantikine Human BAFF/BLyS/TNFSF13B (CAT: PDBLYS0B; R&D Systems, MN, USA) and IL-21 was measured with MAX Deluxe Set Human IL-21 (CAT: 433804; BioLegend, San Diego, CA, USA).

### Data analysis

Data were analyzed using GraphPad Prism v.9. Categorical variables are presented as absolute values. Continued variables are presented as medians with 25th-75th percentiles. Normality tests were performed, and data didn’t have normal distribution. Statistical differences between for 2 groups were calculated with Mann-Whitney U test and for ≥3 groups with Kruskal-Wallis’s test and Dunn’s test as *post hoc*. Correlations were calculated with Spearman test. Statistical significance was represented as follows: ns was p>0.05, *p<0.05, **p<0.01, ***p<0.001 and ****p<0.0001.

## Results

### Demographic and clinical features

Forty-five SLE patients and 15 healthy subjects were included in this study, all females with a median age of 36 (27-46) years for SLE and 32 (21-50) for HS. The clinical features of SLE patients are described in **Table 1**. The median of MexSLEDAI activity index was 2 (0-6) with 55.8% Non-Active, 18.6% LDA and 25.6% HDA SLE patients. This study included newly and previously diagnosed patients with a median of 5 years of disease duration (range 0-30). The most frequent clinical manifestations were hematological (37.8%), mucocutaneous (31%) and renal disorder (24.4%). Three SLE patients were not under treatment at the inclusion moment, while the rest of them were under conventional treatment with prednisone, antimalarial or immunosuppressive drugs.

**Table 1:** Clinical characteristics of SLE patients.

	SLE n=45
Age, years, median (p25 – p75)	36 (27-46)
Gender, (F/M)	45/0
Disease duration, years; median (range)	5 (0-30)
MexSLEDAI, median (p25 – p75)	2 (0-6)
SLEDAI, median (p25 – p75)	2 (0-6)
SLEDAS, median (p25 – p75)	3.19 (0.37-9.93)
BILAG, median (p25 – p75)	1 (0-9)
SLICC-DI, median (p25 – p75)	0 (0-1)
Renal disorder, n (%)	11 (24.4)
Hematological, n (%)	17 (37.8)
Mucocutaneous, n (%)	14 (31)
No treatment, n (%)	3 (6.6)
Antimalarial, n (%)	32 (71.1)
Prednisone, n (%)	20 (44.4)
Prednisone, mg/day, median (p25 – p75)	12.5 (5-36.88)
Azathioprine, n (%)	11 (24.4)
Mycophenolate, n (%)	11 (24.4)
Methotrexate, n (%)	9 (20)
Cyclophosphamide, n (%)	1 (2.2)

Data are shown as median (p25-75) or number of patients and percentage. SLEDAI, Systemic Lupus Erythematosus Disease Index; MexSLEDAI, Mexican version of SLEDAI; SLEDAS, Systemic Lupus Erythematosus Disease Activity Score; BILAG, British Isles Lupus Assessment Group 2004 index; SLICC-DI, Systemic Lupus International Collaborating Clinics.

### CXCR5^-^ CD11c^+^ atypical B cell subpopulations are expanded in SLE patients

**Figure 1:**
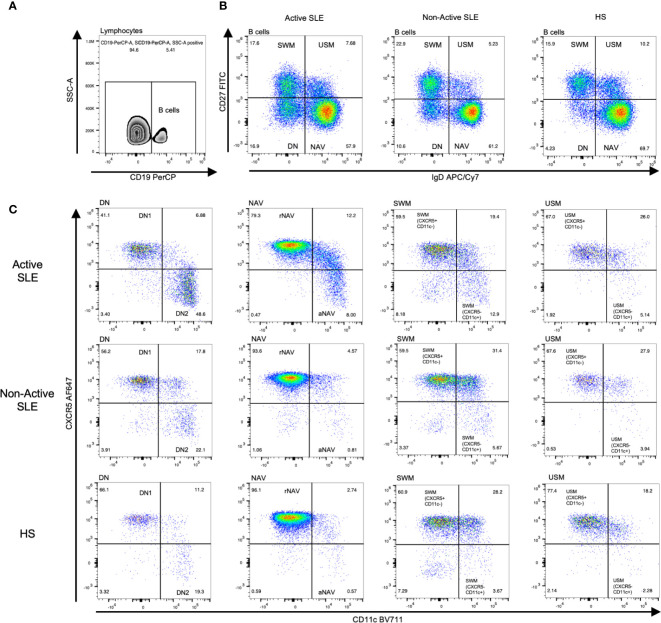
Analysis strategy to identify CXCR5 and CD11c B cell subpopulations. After lymphocyte and single-cell separation, B cells were gated by their CD19^+^ expression **(A)**. B cells of active SLE, Non-Active SLE patients, and HS were subclassified according to their CD27 and IgD expression in DN B cells (CD27^-^ IgD^-^), NAV B cells (CD27^-^ IgD^+^), SWM B cells (CD27^+^ IgD^-^) and USM B cells (CD27^+^ IgD^+^) **(B)**. Each B cell subpopulation was sub-stratified according to CXCR5 and CD11c expression, identifying two general groups: CXCR5^+^ CD11c^-^ B cell subsets (DN1, rNAV, CXCR5^+^ CD11c^-^ SWM, and CXCR5^+^ CD11c^-^ USM) and CXCR5^-^ CD11c^+^ atypical B cell subsets (DN2, aNAV, CXCR5^-^ CD11c^+^ SWM and CXCR5^-^ CD11c^+^ USM) **(C)**. SLE, Systemic Lupus Erythematosus; HS, Healthy subjects; rNAV, resting naïve; aNAV, activated naïve; NAV, Naïve; DN, Double negative; DN1, Double negative 1; DN2, Double negative 2; SWM, Switched memory; USM, Unswitched memory.

Flow cytometry was performed in fresh PBMCs to identify lymphocytes and single cells. Then B cells were identified using the CD19 marker (**Figure 1A**). These B cells were labeled with CD27 and IgD identifying four classic subpopulations: double negative B cells DN (CD27^-^ IgD^-^), naïve B cells NAV (CD27^-^ IgD^+^), switched memory B cells SWM (CD27^+^ IgD^-^) and unswitched memory B cells USM (CD27^+^ IgD^+^) (**Figure 1B**). Afterwards, in order to identify the frequency of the atypical B cell subpopulations we performed a sub-stratification using the CXCR5 and CD11c expression according to the strategy reported by (7). The DN population was separated into DN1 B cells (CXCR5^+^ CD11c^-^) and DN2 B cells (CXCR5^-^ CD11c^+^). The NAV B cells were separated into resting naïve (rNAV) (CXCR5^+^ CD11c^-^) and activated naïve (aNAV) (CXCR5^-^ CD11c^+^). Also, the memory B cells were sub-stratified using the CXCR5 and CD11c expression according to that reported by Sanz et al., 2019, in SWM (CXCR5^+^ CD11c^-^) and SWM (CXCR5^-^ CD11c^+^); USM (CXCR5^+^ CD11c^-^) and USM (CXCR5^-^ CD11c^+^) (**Figure 1C**).

**Figure 2:**
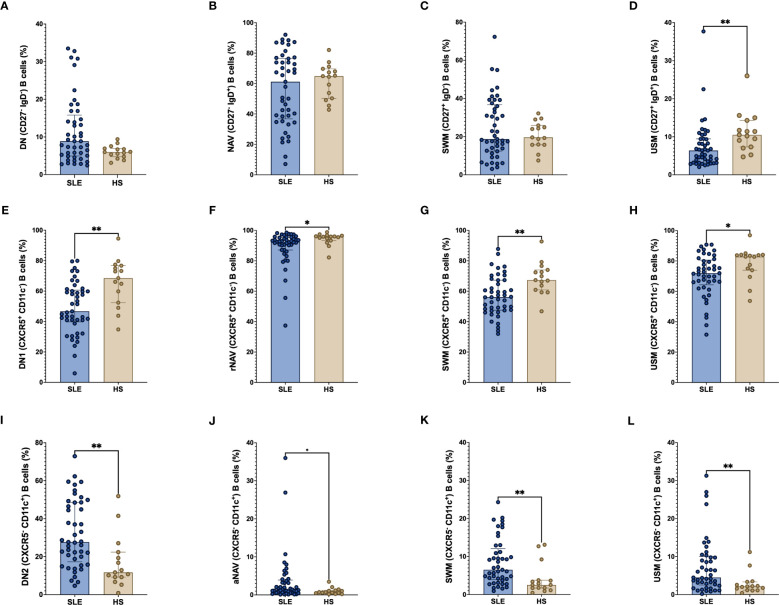
Frequency of CXCR5 and CD11c B cell subpopulations in SLE patients. Frequency of classic B cell subpopulations; DN (CD27^-^ IgD^-^), NAV (CD27^-^ IgD^+^), SWM (CD27^+^ IgD^-^) and USM (CD27^+^ IgD^+^) **(A–D)** in SLE and HS. Frequency of CXCR5^+^ CD11c^-^ B cell subsets (DN1, rNAV, CXCR5^+^ CD11c^-^ SWM, and CXCR5^+^ CD11c^-^ USM) **(E–H)** and CXCR5^-^ CD11c^+^ B cell subsets (DN2, aNAV, CXCR5^-^ CD11c^+^ SWM and CXCR5^-^ CD11c^+^ USM) **(I–L)** between SLE patients and HS. The classic B cell subpopulations were use as parent populations for the CXCR5^+^ CD11c^-^ and CXCR5^-^ CD11c^+^ B cell subsets. SLE, Systemic Lupus Erythematosus; HS, Healthy subject; DN, Double negative; NAV, Naïve; SWM, Switched memory; USM, Unswitched memory; DN1, Double negative 1; rNAV, Resting naïve; DN2, Double negative 2; aNAV, Activated naïve. *p = ≤ 0.05, **p = ≤ 0.01, Mann-Whitney U for two groups comparations.

The classic B cell subpopulations DN, NAV and SWM frequency are similar between SLE patients and HS (**Figure 2A–C**). We found that total USM (CD27^+^ IgD^+^) B cells were decreased in SLE patients compared with HS (median 6.39% vs 10.5%, p = 0.0015) (**Figure 2D**). All the CXCR5^+^ CD11c^-^ B cells which are the more frequent among B cell populations, were decreased in SLE patients: DN1 (46.7% vs 68.5%, p = 0.0012), rNAV (92.5% vs 95.8%, p = 0.0171), CXCR5^+^ CD11c^-^ SWM (56.1% vs 67.3%, p = 0.0029) and CXCR5^+^ CD11c^-^ USM (71.9% vs 82.8%, p = 0.0484) (**Figures 2E–H**). In contrast, the CXCR5^-^ CD11c^+^ atypical B cell subsets were increase in SLE patients in comparison with HS: DN2 (27.7% vs 11.7%, p = 0.0013), aNAV (1.4% vs 0.7%, p = 0.0383), CXCR5^-^ CD11c^+^ SWM (6.5% vs 2.5%, p = 0.0014) and CXCR5^-^ CD11c^+^ USM (4.5% vs 2.1%, p = 0.0069) (**Figures 2I–L**).

### Differential expression of BAFF system on classic B cell subpopulations in SLE patients

**Figure 3:**
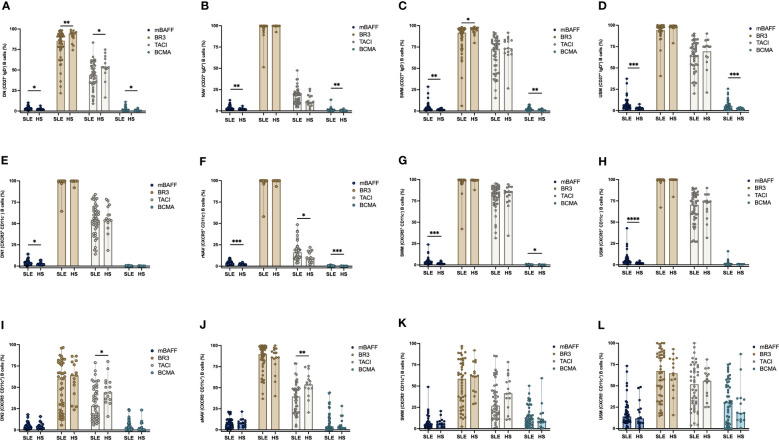
Frequency expression of mBAFF, BR3, TACI, and BCMA in B cell subpopulations between SLE patients and HS. Expression of mBAFF, BR3, TACI, and BCMA in DN (CD27- IgD-), NAV (CD27^-^ IgD^+^), SWM (CD27^+^ IgD^-^) and USM (CD27^+^ IgD^+^) B cells in SLE patients and HS **(A–D)**. Expression of mBAFF, BR3, TACI, and BCMA in CXCR5^+^ CD11c^-^ B cell subsets (DN1, rNAV, CXCR5^+^ CD11c^-^ SWM, and CXCR5^+^ CD11c^-^ USM) **(E–H)**. Expression of mBAFF, BR3, TACI, and BCMA in CXCR5^-^ CD11c^+^ atypical B cell subsets (DN2, aNAV, CXCR5^-^ CD11c^+^ SWM and CXCR5^-^ CD11c^+^ USM) **(I–L)** in SLE patients and HS. SLE, Systemic Lupus Erythematosus; HS, Healthy subject; DN, Double negative; NAV, Naïve; SWM, Switched memory; USM, Unswitched memory; DN1, Double negative 1; rNAV, Resting naïve; DN2, Double negative 2; aNAV, Activated naïve; mBAFF, membrane B cell activating factor; BR3, B cell activating factor receptor; TACI, Transmembrane activator calcium modulator and cyclophilin ligand interactor; BCMA, B cell maturation antigen. *p = ≤0.05, **p = ≤0.01, ***p = ≤0.001, ****p = ≤0.0001, Mann-Whitney U for two group comparations.

The expression of mBAFF, BR3, TACI and BCMA were measured in each B cell subpopulation and compared between SLE patients and HS. A higher frequency of mBAFF was observed in the classic B cell subpopulations DN, NAV, SWM and USM in SLE patients than HS (DN: 2.85% vs 2.29%, p = 0.0233; NAV: 2.7% vs 1.9%, p = 0.0044; SWM: 2.6% vs 1.3%, p = 0.0044; and USM: 5.3% vs 2.9%; p = 0.0002). Also, the frequency of BCMA positive B cells were elevated in SLE patients (DN: 1.08% vs 0.40%, p = 0.0400; NAV: 0.79% vs 0.27%, p = 0.0147; SWM: 1.7% vs 0.7%, p = 0.0054; and USM: 4.2% vs 2.0%, p = 0.0079) (**Figures 3A–D**). Whereas the DN and SWM B cells had lower expression of BR3 in SLE patients compared to HS (DN: 85.6% vs 94.4%, p = 0.0077 and SWM: 91.8% vs 95.8%, p = 0.0142 respectively) (**Figures 3A, C**). The expression of TACI was lower in DN B cells of SLE patients (43.6% vs 53.3%, p = 0.0212) (**Figure 3A**).

### Increase expression of mBAFF in CXCR5^+^ CD11c^-^ B cell subsets in SLE patients

The frequency of mBAFF positive B cells was higher in SLE patients compared with HS in the CXCR5^+^ CD11c^-^ B cell subsets: DN1 (2.98% vs 2.04%, p = 0.0386), rNAV (3.12% vs 1.71%, p = 0.0009), CXCR5^+^ CD11c^-^ SWM (2.87% vs 1.23%, p = 0.0005) and CXCR5^+^ CD11c^-^ USM (4.16% vs 1.90%, p = < 0.0001) (**Figures 3E–H**). BCMA was increased in the B cell subsets of SLE patients: rNAV (0.21% vs 0.07%, p = 0.0009) and CXCR5^+^ CD11c^-^ SWM (0.24% vs 0.15%, p = 0.0288) compared to HS (**Figures 3F, G**). The BCMA expression by MFI was lower in rNAV (355 vs 463, p = 0.0012) and CXCR5^+^ CD11c^-^ USM (390 vs 546, p = 0.0039) of SLE patients (**Supplementary Figures 2F, H**). TACI was also increased in rNAV B cells from SLE patients compared to HS (16.2% vs 8.86%, p = 0.0162) (**Figure 3F**).

### DN2 and aNAV B cells have a decreased expression of TACI in SLE patients

The CXCR5^-^ CD11c^+^ atypical B cells DN2 and aNAV are described as ABC cells with an important role in the development of SLE for their capacity to develop into antibody-secreting cells. We found that these subpopulations have a decreased expression of TACI in SLE patients compared to HS, DN2 (28.25% vs 44.0%, p = 0.0153) and aNAV (39.55% vs 53.8%, p = 0.0020) (**Figures 3I, J**). The expression of mBAFF, BR3, TACI and BCMA in the atypical SWM (CXCR5^-^ CD11c^+^) and USM (CXCR5^-^ CD11c^+^) B cells (**Figures 3K, L**) was similar between SLE patients and HS. The expression of BCMA on aNAV was decreased in SLE patients compared with HS (MFI= 1,939 vs 4,075, p = 0.0315) (**Supplementary Figure 2J**).

### sBAFF and IL-21 are correlated with atypical B cells DN2 and aNAV

**Figure 4:**
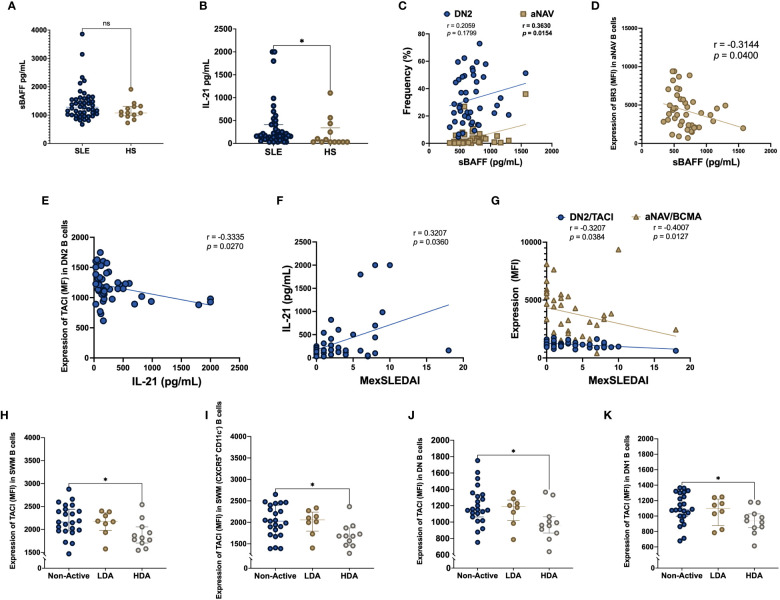
sBAFF and IL-21 serum levels in SLE patients, HS and according to disease activity Serum levels of soluble BAFF (sBAFF) and IL-21 were measured in SLE patients and HS **(A, B)**. Correlation between sBAFF and the frequency of DN2 and aNAV B cells **(C)**. Correlation between sBAFF and BR3 MFI expression in aNAV B cells **(D)**. Soluble IL-21 correlation with TACI MFI expression in DN2 B cells **(E)**. Correlation between soluble IL-21 and MexSLEDAI activity index **(F)**. Correlation among MexSLEDAI and the MFI expression of TACI in DN2 and BCMA in aNAV B cells **(G)**. TACI MFI expression in SLE patients according to disease activity measured with MexSLEDAI index in SWM **(H)**, CXCR5^+^ CD11c^-^ SWM **(I)**, DN **(J)** and DN1 **(K)** B cells. MexSLEDAI values: Non-Active (0-2), LDA (3-5) and HDA (≥6). SLE: Systemic Lupus Erythematosus, HS, Healthy subjects; DN2, Double negative 2; aNAV, Activated naïve; sBAFF, soluble B cell activating factor; IL-21, Interleukin 21; BR3, B cell activating factor receptor; TACI, Transmembrane activator calcium modulator and cyclophilin ligand interactor; BCMA, B cell maturation antigen; MFI, Geometric-Mean Fluorescence Intensity; LDA, Low disease activity; HDA, High disease activity. Spearman rank test was used for correlations, Mann-Whitney U for two group comparations, Kruskal Wallis and Dunn’s *post hoc* for three group comparations *p = ≤0.05, **p = ≤0.01; ns, non-significant p-value.

Soluble levels of BAFF and IL-21 were compared in SLE patients and HS. Although sBAFF didn’t show differences between SLE patients and HS (1281 vs 1076 pg/mL, p = 0.1322), soluble levels of IL-21 were higher in SLE patients compared to HS (161 vs 31 pg/mL, p = 0.0388) (**Figures 4A, B**). Spearman correlation test was made for sBAFF and IL-21 with the frequency of the B cell subpopulations and the BAFF system expression. sBAFF showed a positive correlation with the frequency of aNAV B cells (r = 0.3630, p = 0.0154) (**Figure 4C**) and negative correlation with the expression (MFI) of BR3 in aNAV B cells (r = -0.3144, p = 0.0400) (**Figure 4D**). Additionally, IL-21 had a negative correlation with the expression (MFI) of TACI in DN2 B cells (r = -0.3335, p = 0.0270) (**Figure 4E**). Furthermore, there was a positive correlation of IL-21 with the MexSLEDAI score (r = 0.3207, p = 0.0360) (**Figure 4F**).

### Decreased expression of TACI is associated with higher disease activity

MexSLEDAI score was negatively correlated with the expression (MFI) of TACI in DN2 B cells (r = -0.3207, p = 0.0384) and BCMA in aNAV B cells (r = -0.4007, p = 0.0127) (**Figure 4G**). The expression of the BAFF system in the B cell subpopulations was compared among SLE patients stratified according to MexSLEDAI score. The expression of TACI (MFI) was decreased in active SLE patients compared with Non-Active patients in SWM B cells (1816 vs 2250, p = 0.0187), CXCR5^+^ CD11c^-^ SWM B cells (1680 vs 2126, p = 0.0113), DN B cells (961 vs 1225, p = 0.0205) and DN1 B cells (938 vs 1122, p = 0.0252) (**Figures 4H–K**).

The frequency of atypical B cell subsets DN2, CXCR5^-^ CD11c^+^ SWM and CXCR5^-^ CD11c^+^ USM B cells is correlated with the expression of the BAFF system in SLE patients.

**Figure 5:**
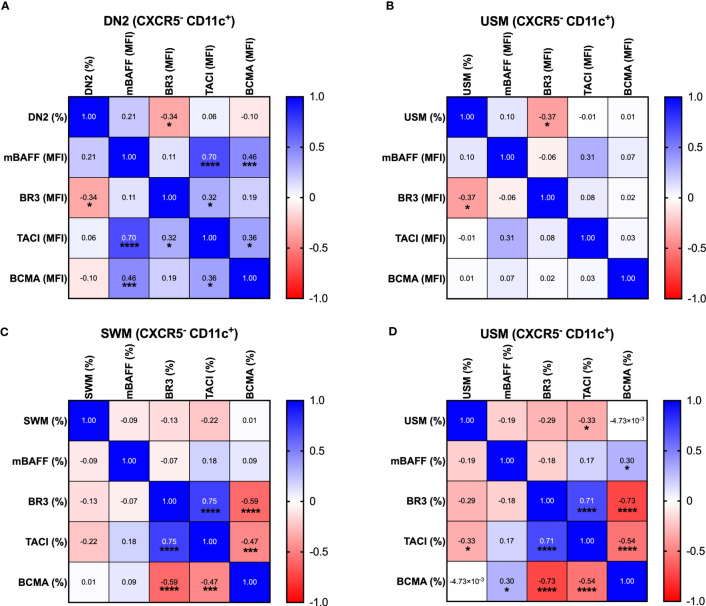
Correlation among mBAFF, BR3, TACI, BCMA and the B cell subpopulations Correlation between the MFI expression of mBAFF, BR3, TACI and BCMA with the frequency of DN2 B cells **(A)**. Correlation between the MFI expression of mBAFF, BR3, TACI and BCMA with the frequency of USM (CXCR5^-^ CD11c^+^) B cells **(B)**. Correlation between the percentage expression of mBAFF, BR3, TACI and BCMA with SWM (CXCR5^-^ CD11c^+^) B cells **(C)**. Correlation between the percentage expression of mBAFF, BR3, TACI and BCMA with USM (CXCR5^-^ CD11c^+^) B cells **(D)**. DN2, Double negative 2; mBAFF, membrane B cell activating factor; BR3, B cell activating factor receptor; TACI, Transmembrane activator calcium modulator and cyclophilin ligand interactor; BCMA, B cell maturation antigen; MFI, Geometric-Mean Fluorescence Intensity; Spearman rank test was used for correlations. *p = ≤0.05, ***p = ≤0.001, ****p = ≤0.0001.

We evaluated correlations among mBAFF, BR3, TACI and BCMA MFI expression and the frequency of DN2 B cells (**Figure 5A**) and CXCR5^-^ CD11c^+^ USM B cells (**Figure 5B**). Also, correlations among the percentage’s expression of mBAFF, BR3, TACI and BCMA and the frequency of CXCR5^-^ CD11c^+^ SWM B cells (**Figure 5C**) and CXCR5^-^ CD11c^+^ USM B cells (**Figure 5D**) were analyzed. A negative correlation between the frequency of DN2 and CXCR5^-^ CD11c^+^ USM B cells with the MFI expression of BR3 was observed. Also, we found a negative correlation between the frequency of CXCR5^-^ CD11c^+^ SWM and CXCR5^-^ CD11c^+^ USM B cells with the percentage’s expression of TACI.

## Discussion

SLE is an autoimmune systemic disease, characterized by autoantibody production and diverse clinical manifestations. The role of the BAFF system has been associated with the pathogenesis of SLE and disease activity by its capacity to promote B cell survival and proliferation. Recently, CD11c^+^ B cells have been described as potential self-reactive B cells prone to develop into ASC in an extra-follicular manner. Jenks et al. has characterized some of these B cell subpopulations by the markers CXCR5^-^ CD11c^+^ (7). Our work aims to analyze the BAFF system expression in the CXCR5^-^ CD11c^+^ atypical B cells in order to elucidate its role in the SLE pathogenesis.

Firstly, we found a decreased frequency of unswitched memory B cells (CD27^+^ IgD^+^) in SLE patients. This decreased USM frequency was reported to be related with the increase of DN B cells (CD27^-^ IgD^-^) after TLR9 stimulation (13). Although we found an increase number of DN B cells, the difference wasn’t significant as reported in previous studies (13, 21). Like ABCs, the CD27^+^ CD21^-^ memory B cells have been characterized before in malaria-exposed donors as “activated memory B cells” with high expression of Blimp-1 and IRF4 (22). As expected, the CXCR5^+^ CD11c^-^ B cell subsets have the highest frequency in all B cell subpopulations. However, we found a decreased frequency of these CXCR5^+^ CD11c^-^ B cell subsets in SLE patients. The above could be related with the elevated frequency of CXCR5^-^ CD11c^+^ B cells in SLE patients, which correspond to atypical B cells that resemble autoimmunity-associated B cells (ABCs). ABCs have been described previously by the expression of CD11c and T-bet (8, 11), afterward by absence of CXCR5 marker as: CXCR5^-^ CD11c^+^ atypical DN2 and aNAV B cells (7, 23).

On the other hand, we found an increased expression of mBAFF in the classic B cell subpopulations (DN, NAV, SWM and USM) as well as in all the CXCR5^+^ CD11c^-^ B cell subsets (DN1, rNAV, CXCR5^+^ CD11c^-^ SWM and USM B cells). This elevated expression of mBAFF could be a consequence of B cells activation related to SLE pathogenesis. The expression of membrane-bound BAFF (mBAFF) is null in resting B cells; however, *in vitro* studies have shown that B cells can express mBAFF when they are activated by TLR9 through MyD88 (24, 25). B cells of SLE have higher expression of TLR9 (26) and its effect is related with the B cells activation and extrafollicular subpopulations development (27). CXCR5^-^ CD11c^+^ atypical B cell subsets also had elevated expression of mBAFF compared with CXCR5^+^ CD11c^-^ B cells, in both SLE and HS patients, suggesting the importance of this cytokine in the maintenance and likely in ABCs development.

The low expression of BR3 on B cells has been reported previously in SLE patients and even the lower BR3 expression was correlated with the disease activity (6, 28). In this study we found a decreased expression of BR3 on DN and SWM B cells of SLE patients. It is known that BR3 can be shed by ADAM10 as consequence of BAFF binding and enhanced by TLR9 activation in the presence of TACI (29). Therefore, the low expression of BR3 in these cells in part could be explained by the release of soluble BR3 (sBR3) (30). Other studies have demonstrated that the decreased expression of BR3 measured by flow cytometry could be associated to the receptor occupancy (31). Also, in ABCs (CD11c^+^ T-bet^+^), a lower BR3 expression has been previously reported (8). In this study, we also found a decreased expression of BR3 in ABCs although it didn’t show significant differences. Another study carried out in mice report an ABC subset (CD21^-^ CD23^-^) that have similar expression of BR3 with the follicular B cells and have the same capacity to bind BAFF but doesn’t rely on the BAFF/BR3 axis to survive (16). This could reflect an increase in the ABCs subpopulation at the expense of conventional follicular B cells frequency. This is interesting to analyze, since it could reflect a phenomenon of competition for niches, in which ABCs sequester the available BAFF by being more avid. The remarkable low expression of BR3 in atypical B cell subpopulations in addition to the increased frequency of these cells in SLE could explain the decreased expression of BR3 on CD19^+^ cells in SLE patients reported previously (6, 28, 31, 32).

In addition, our work showed that SLE patients had an increased expression of BCMA on rNAV, CXCR5^+^ CD11c^-^ SWM and in the classic B cell subpopulations (DN, NAV, SWM and USM B cells). The elevated expression of BCMA on B cells have been reported in previous studies. Also, this elevated expression of BCMA could be related with TLR9 stimulation (25, 33). In this study the expression of BCMA was found to increase in CXCR5^-^ CD11c^+^ atypical B cells with no differences between SLE and HS. This increased expression of BCMA on CD11c^+^ B cells could be associated with the capacity of BCMA to promote Ag-presentation through JNK-axis activation (34). T follicular helper cells (Tfh) have been associated with CD11c^+^ B cells because of their interrelationship with IL-21, nevertheless, it has been hypothesized that CD11c^+^ B cells can stimulate Tfh cells through strong Ag-presentation (35, 36). Although in the present study we observe a higher percentage of B cells positive for BCMA, some subpopulations of SLE patients showed a lower expression of BCMA by MFI. Particularly for aNAV in which a negative correlation between BCMA expression and disease activity was found. This finding had been previously reported in our research group (6).

Furthermore, we found an increased expression of TACI on rNAV B cells of SLE patients. However, the CXCR5^-^ CD11c^+^ atypical B cells DN2 and aNAV, showed decreased expression of TACI in SLE patients compared with HS. This finding is interesting because of the paradoxical functions of TACI in B cells (37). It’s known that TACI can promote Ig-class switching in T-independent responses (38), but the decreased or null expression of TACI is associated with expanded numbers of B cells (39). In addition, TACI can induce and maintain Blimp-1 expression (40) that promotes B cell differentiation instead of proliferation which emphasizes the control exerted by TACI in the mature B cells pool, positioning TACI as an important receptor for the B cell homeostasis. It is possible that the interaction of CD11c^+^ B cells with Tfh-like cells could be mediated by ICOS-ICOSL because TACI deficiency can increase ICOSL expression on GC B cells in mice (36, 41). The immunoregulatory effect of TACI has been tested in mice, describing that TACI can upregulate the expression of Fas and FasL promoting apoptosis in MZ B cells (42). Although TACI is important in the B cell maturation, loss of TACI has been associated with autoimmunity development in mice (43). Also, in humans TACI deficiency was associated with breached immune tolerance in subjects with common variable immune deficiency (44). We hypothesize that the lower expression of TACI in the CXCR5^-^ CD11c^+^ atypical B cells DN2 and aNAV in SLE could be responsible in part for loss of regulation of these populations, highlighting the BAFF role in the SLE pathogenesis.

Finally, levels of sBAFF were positively correlated with aNAV frequency, whereas the expression of BR3 on aNAV B cells was negatively correlated with sBAFF levels. This correspond with previous reports where the decreased expression of BR3 was correlated with elevated levels of sBAFF (6, 28, 30). Soluble levels of IL-21 were elevated in SLE and correlated with the disease activity as previously reported (45). It has been described that the atypical B cells are developed through IL-21 signaling (8) in addition to that IL-21 can suppress the expression of TACI in activated B cells (46). This relation could explain that the soluble levels of IL-21 were negatively correlated with the expression of TACI on DN2 B cells. On the other hand, the decreased expression of TACI could be related with the release of the receptor as recently Hoffmann et al. has demonstrated that TACI is shed constitutively by the metalloprotease ADAM10 and the soluble form of TACI acts as a decoy receptor that reduces the NF-κB activation and thus promotes a negative effect on signaling (47). In the context of TACI occupancy, the evidence shows the BAFF 3-mer affinity for TACI is poor compared with the BAFF oligomeric forms (48). Since the prevalent soluble BAFF form is the 3-mer we hypothesize the preferential binding of this form occurs with BR3 *in vivo*.

We previously have reported that the decreased expression of TACI and BCMA on B cells was related with the disease activity in SLE (6). In this study we found that the expression of TACI on DN2 and BCMA on aNAV B cells were negatively correlated with the disease activity. Furthermore, we found that HDA patients has a decreased (MFI) expression of TACI compared with Non-Active SLE patients in SWM, CXCR5^+^ CD11c^-^ SWM, DN and DN1 B cells. The above highlights the regulatory role of TACI in B cells homeostasis, which appears to be affected in the autoimmunity context.

## Conclusion

These results suggest a participation of the BAFF system in CXCR5^-^ CD11c^+^ atypical B cell subsets in SLE patients. Decreased TACI expression on DN2 B cells correlated with high disease activity in SLE patients supporting the immunoregulatory role of TACI in autoimmunity.

## Data availability statement

The raw data supporting the conclusions of this article will be made available by the authors, without undue reservation.

## Ethics statement

The studies involving humans were approved by The ethics in research committee of the Hospital General de Occidente, Guadalajara, Mexico. The studies were conducted in accordance with the local legislation and institutional requirements. The participants provided their written informed consent to participate in this study.

## Author contributions

JG, DS-C and CP-S contributed with conception of the study. JG, IR-F, PO-L and CP-S contributed with the design of the experiments. MM-R and NS-F contributed with patients’ inclusion. JG and IR-F done data acquisition. JG, DS-C, NE-G and PO-L contributed with data analysis and interpretation. JG wrote the first draft of the manuscript. DS-C, AC, JM-V and CP-S wrote sections of the manuscript. All authors contributed to the article and approved the submitted version.

## Supplementary material

Supplementary Figure 1 Representative fluorescence minus one (FMO) control and Autofluorescence (AF) controls The BAFF system members were measured using antibodies with PE. FMO control was used for TACI, BR3 and mBAFF in order to delimitate positive events properly **(A)**. For the appropriate gating of BCMA, the AF was used as a negative control since it showed a good separation of positive events **(B)**. rNAV, resting naïve; aNAV, activated naïve; NAV, Naïve; DN, Double negative; DN1, Double negative 1; DN2, Double negative 2; SWM, Switched memory; USM, Unswitched memory; mBAFF, membrane B cell activating factor; BR3, B cell activating factor receptor; TACI, Transmembrane activator calcium modulator and cyclophilin ligand interactor; BCMA, B cell maturation antigen; FMO, fluorescence minus one; AF, Autofluorescence.

Supplementary Figure 2 Expression of mBAFF, BR3, TACI, and BCMA by MFI in DN (CD27- IgD-), NAV (CD27^-^ IgD^+^), SWM (CD27^+^ IgD^-^) and USM (CD27^+^ IgD^+^) B cells in SLE patients and HS **(A–D)**. Expression of mBAFF, BR3, TACI, and BCMA in CXCR5^+^ CD11c^-^ B cell subsets (DN1, rNAV, CXCR5^+^ CD11c^-^ SWM, and CXCR5^+^ CD11c^-^ USM) **(E–H)**. Expression of mBAFF, BR3, TACI, and BCMA in CXCR5^-^ CD11c^+^ atypical B cell subsets (DN2, aNAV, CXCR5^-^ CD11c^+^ SWM and CXCR5^-^ CD11c^+^ USM) **(I–L)** in SLE patients and HS. MFI, geometric mean fluorescence intensity; SLE, Systemic Lupus Erythematosus; HS, Healthy subject; DN, Double negative; NAV, Naïve; SWM, Switched memory; USM, Unswitched memory; DN1, Double negative 1; rNAV, Resting naïve; DN2, Double negative 2; aNAV, Activated naïve; mBAFF, membrane B cell activating factor; BR3, B cell activating factor receptor; TACI, Transmembrane activator calcium modulator and cyclophilin ligand interactor; BCMA, B cell maturation antigen. *p = ≤0.05, **p = ≤0.01, Mann-Whitney U for two group comparisons.
