# Supplementary material for: BAFF system expression in double negative 2, activated naïve and activated memory B cells in systemic lupus erythematosus
Source: Front Immunol. 2023 Aug 22;14:1235937. doi: 10.3389/fimmu.2023.1235937 (PMC10478082; doi:10.3389/fimmu.2023.1235937)
Supplement: Supplementary file 1 [file Table_1.docx]

| Specificity | Fluorochrome | Clone | Concentration | CAT | Company |
| --- | --- | --- | --- | --- | --- |
| CD19 | PerCP | HIB19 | 100 ug/mL | 302227 | BioLegend Inc, San Diego, CA, USA |
| CD27 | FITC | O323 | 100 ug/mL | 302806 | BioLegend Inc, San Diego, CA, USA |
| IgD | APC/Cy7 | IA6-2 | 200 ug/mL | 348217 | BioLegend Inc, San Diego, CA, USA |
| CD185/CXCR5 | AF647 | RF8B2 | 200 ug/mL | 558113 | BD Biosciences, San Jose CA, USA |
| CD11c | BV711 | B-lY6 | 200 ug/mL | 563130 | BD Biosciences, San Jose CA, USA |
| CD257/BAFF | PE | T7-241 | 400 ug/mL | 318606 | BioLegend Inc, San Diego, CA, USA |
| CD268/BR3 | PE | 11C1 | 50 ug/mL | 316905 | BioLegend Inc, San Diego, CA, USA |
| CD267/TACI | PE | 1A1 | 200 ug/mL | 311906 | BioLegend Inc, San Diego, CA, USA |
| CD269/BCMA | BV421 | 19F2 | 150 ug/mL | 357520 | BioLegend Inc, San Diego, CA, USA |

**Supplementary table 1. Antibodies for flow cytometry**
