# Supplementary figures and images for: BAFF system expression in double negative 2, activated naïve and activated memory B cells in systemic lupus erythematosus
Source: Front Immunol. 2023 Aug 22;14:1235937. doi: 10.3389/fimmu.2023.1235937 (PMC10478082; doi:10.3389/fimmu.2023.1235937)

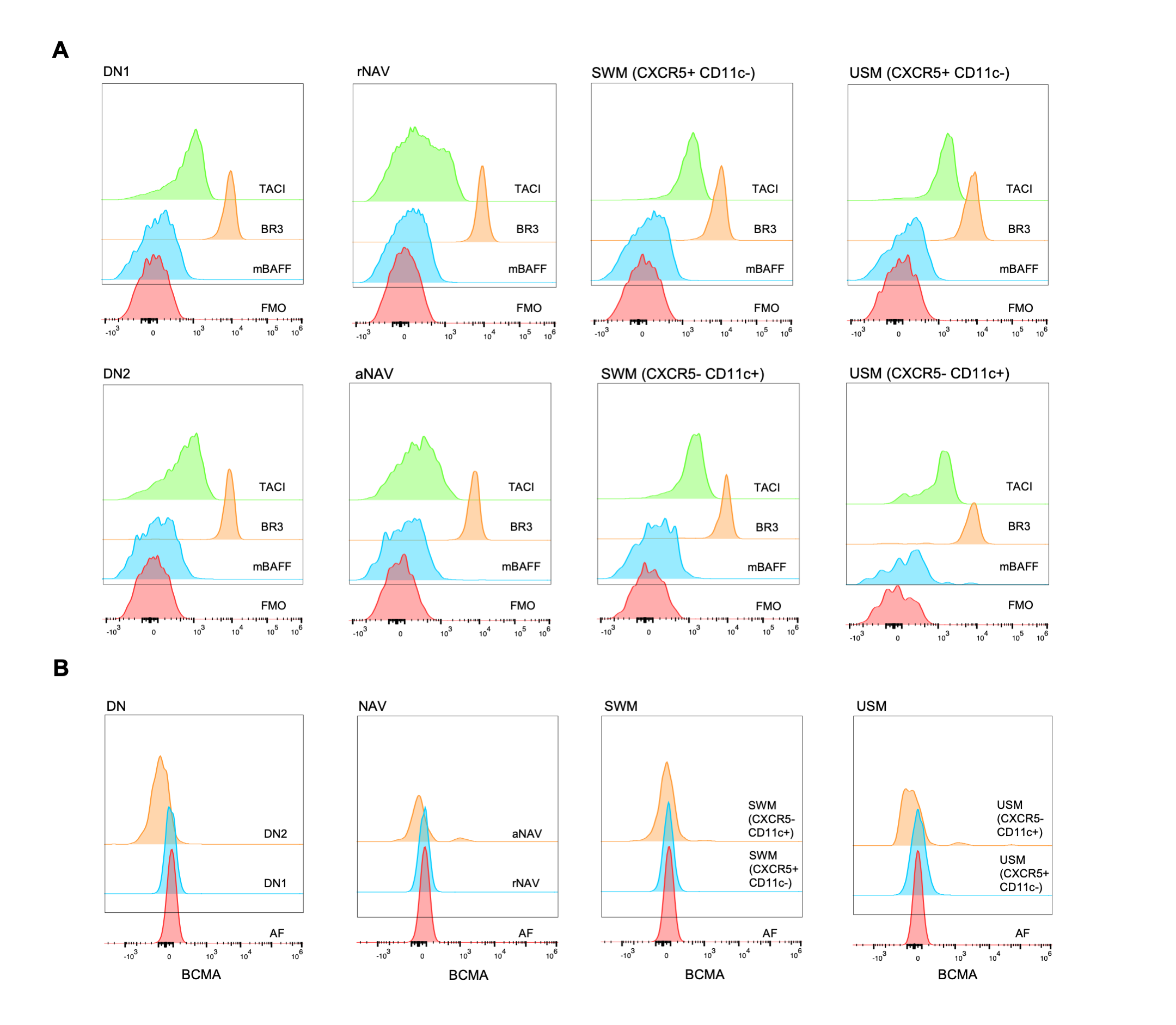

Supplement: Supplementary Figure 1 — Representative fluorescence minus one (FMO) control and Autofluorescence (AF) controls The BAFF system members were measured using antibodies with PE. FMO control was used for TACI, BR3 and mBAFF in order to delimitate positive events properly (A). For the appropriate gating of BCMA, the AF was used as a negative control since it showed a good separation of positive events (B). rNAV, resting naïve; aNAV, activated naïve; NAV, Naïve; DN, Double negative; DN1, Double negative 1; DN2, Double negative 2; SWM, Switched memory; USM, Unswitched memory; mBAFF, membrane B cell activating factor; BR3, B cell activating factor receptor; TACI, Transmembrane activator calcium modulator and cyclophilin ligand interactor; BCMA, B cell maturation antigen; FMO, fluorescence minus one; AF, Autofluorescence. [file Image_1.tiff]

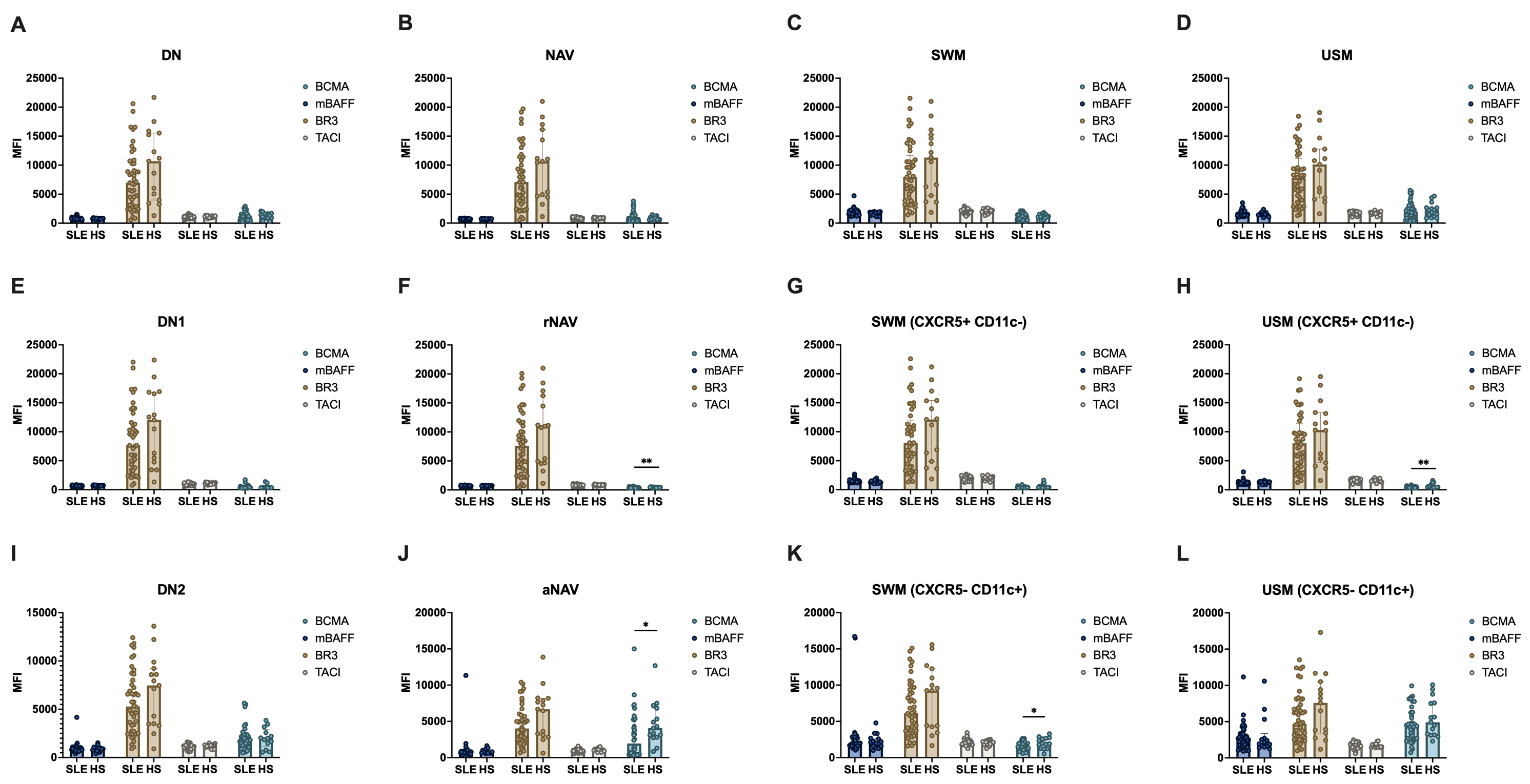

Supplement: Supplementary Figure 2 — Expression of mBAFF, BR3, TACI, and BCMA by MFI in DN (CD27- IgD-), NAV (CD27- IgD+), SWM (CD27+ IgD-) and USM (CD27+ IgD+) B cells in SLE patients and HS (A–D). Expression of mBAFF, BR3, TACI, and BCMA in CXCR5+ CD11c- B cell subsets (DN1, rNAV, CXCR5+ CD11c- SWM, and CXCR5+ CD11c- USM) (E–H). Expression of mBAFF, BR3, TACI, and BCMA in CXCR5- CD11c+ atypical B cell subsets (DN2, aNAV, CXCR5- CD11c+ SWM and CXCR5- CD11c+ USM) (I–L) in SLE patients and HS. MFI, geometric mean fluorescence intensity; SLE, Systemic Lupus Erythematosus; HS, Healthy subject; DN, Double negative; NAV, Naïve; SWM, Switched memory; USM, Unswitched memory; DN1, Double negative 1; rNAV, Resting naïve; DN2, Double negative 2; aNAV, Activated naïve; mBAFF, membrane B cell activating factor; BR3, B cell activating factor receptor; TACI, Transmembrane activator calcium modulator and cyclophilin ligand interactor; BCMA, B cell maturation antigen. *p = ≤0.05, **p = ≤0.01, Mann-Whitney U for two group comparisons. [file Image_2.tiff]
